# Biosynthesis of copper carbonate nanoparticles by ureolytic fungi

**DOI:** 10.1007/s00253-017-8451-x

**Published:** 2017-08-10

**Authors:** Qianwei Li, Geoffrey Michael Gadd

**Affiliations:** 1 Geomicrobiology Group, School of Life Sciences, University of Dundee, Dundee, , DD1 5EH Scotland UK; 2 State Key Laboratory of Heavy Oil Processing, Beijing Key Laboratory of Oil and Gas Pollution Control, China University of Petroleum, 18 Fuxue Road, Changping District, Beijing, 102249 People’s Republic of China

**Keywords:** Ureolytic fungi, Copper carbonate, Extracellular protein, Biosynthesis

## Abstract

In this research, the ureolytic fungi *Neurospora crassa*, *Pestalotiopsis* sp. and *Myrothecium gramineum* were investigated for the preparation of nanoscale copper carbonate and the role of fungal extracellular protein in such mineral formation. After incubation in urea-modified media, carbonate-laden fungal supernatants were used for the precipitation of copper carbonate, with experimental results agreeing closely with those obtained using geochemical modelling (Geochemist’s Workbench). Compared with commercial and chemically synthesized copper carbonate, the minerals obtained using fungal supernatants were nanoscale and showed varying morphologies. It was found that extracellular protein played an important role in determining the size and morphology of the carbonate minerals precipitated, and after mixture with CuCl_2_ and resultant copper carbonate precipitation, more than 80% protein was removed from the *N. crassa* supernatant. Moreover, with addition of extracellular protein extracted from different fungal supernatants or standard bovine serum albumin, more than 96% of protein was removed by carbonate mineral precipitation. These results provide direct experimental evidence for the preparation of copper carbonate nanoparticles utilizing fungal ureolytic activity and show that fungal extracellular protein plays an important role in the formation and size of specific nano metal carbonates. Such a process provides opportunities for production of specific and/or novel metal carbonate nanoparticles of applied relevance, and as precursors of other useful biomineral products such as oxides.

## Introduction

Nanotechnology refers to the synthesis and exploitation of materials with at least one dimension in the nanometre range (≤ 100 nm) (Bhattacharya and Gupta 2005). Due to their novel optical (Królikowska et al. 2003), chemical (Kumar et al. 2003), photoelectrochemical (Chandrasekharan and Kamat 2000) and electronic (Pető et al. 2002) properties, much research has been carried out on the synthesis of nanomaterials including chemical composition, size and attainment of high monodispersity (Mandal et al. 2006). The synthesis of nanoparticles by chemical methods shows some advantages for large productivity within a relatively short time by adjusting the concentrations of reacting chemicals and controlling the reaction environment, while disadvantages include the high energy demand, cost and the use of extra toxic chemicals (Murray et al. 2000). Physical methods for nanoparticle production, e.g. sputter deposition, laser ablation, and cluster bean deposition, are often difficult (Gade et al. 2010). The development of reliable and environmentally friendly technologies for the synthesis of nanomaterials has therefore become an important aspect of nanotechnology.

It is well known that many microorganisms, including bacteria, yeasts and other fungi, are able to produce inorganic materials intracellularly or extracellularly at nanoscale and microscale (Bhattacharya and Gupta 2005; Gadd 2004, 2010; Li et al. 2011). Salunke et al. (2015) compared the abilities of *Saccharophagus degradans* and *Saccharomyces cerevisiae* for the synthesis of manganese dioxide nanoparticles, and showed that *S. cerevisiae* could provide a simple and reliable approach for the production of this nanomaterial. The synthesis of gold nanoparticles has received considerable attention due to their unique and tunable surface plasmon resonance (SPR) (El-Sayed 2001) and their applications in biomedical science including drug delivery, tissue/tumour imaging, photothermal therapy and immunochromatographic identification of pathogens in clinical specimens (Huang 2006). He et al. (2007) demonstrated that gold nanoparticles (10–20 nm) were formed at pH 7 by the bacterium *Rhodopseudomonas capsulata* based on the bioreduction of AuCl_4_^−^. A lead-tolerant marine yeast strain *Rhodosporidium diobovatum* was used for the synthesis of lead sulphide nanoparticles, and it was found that the metallic PbS particles were formed intracellularly with a size range of 2–5 nm (Seshadri et al. 2011). Compared with bacteria and yeasts, the use of filamentous fungi for the synthesis of nanoparticles seems more promising since they are easily cultured under a controlled environment and can secrete large amounts of extracellular enzymes which are involved in nanomaterial synthesis (Mandal et al. 2006; Bansal et al. 2011).

Fungi can accumulate metals by a number of mechanisms including biosorption, complexation to metabolites and polymers, binding to specific polypeptides and metabolism-dependent accumulation (Gadd 2007; Gade et al. 2010). Another broad mechanism of metal immobilization is through formation of elemental forms or biominerals by means of various redox transformations or excretion of metabolites (Gadd 2010). Most attention to date in the microbial context has focussed on elemental forms, especially silver. Extracellular synthesis of silver nanoparticles (5–45 nm) was achieved using the fungus *Fusarium oxysporum* when 10 g (wet weight) of biomass was exposed to an aqueous silver solution containing 1 mM Ag^+^: the particles were strongly stabilized by secreted proteins (Ahmad et al. 2003). Fungal supernatants from *Penicillium brevicompactum* WA 2315 cultures were also applied for the synthesis of silver nanoparticles, and the possibility of protein as a stabilizing material in the nanoparticles was revealed by Fourier transform infrared spectroscopy (FTIR) analysis (Shaligram et al. 2009). Several reports have pointed out the importance of extracellular proteins in the biosynthesis of nanoparticles, which has indicated an alternative means for their large-scale biosynthesis. Bansal et al. (2004) demonstrated that extracellular proteins, secreted by *F. oxysporum*, with a molecular weight around 24 to 28 kDa, were responsible for the biosynthesis of zirconia nanoparticles. Although much research has been carried out on the biosynthesis of metal nanoparticles (e.g. Au, Ag), and magnetic and non-magnetic oxide and sulphide nanoparticles, there is little knowledge about the biosynthesis of metal carbonate nanominerals and their potential. Metal carbonates can be a good precursor for preparation of metal oxides, especially for nanoscale transition metal oxides which are excellent candidates for electrode materials (Poizot et al. 2000; Dillon et al. 2008; Wu et al. 2012; Li et al. 2016). Copper oxide has attracted attention due to its diverse applications in gas sensors, catalysis, batteries, high-temperature superconductors, solar energy conversion and field emission emitters (Cava 1990; Tranquada et al. 1995; Ren et al. 2009). The ability of ureolytic microorganisms to precipitate carbonates is a well-known phenomenon. Such microbially-induced carbonate precipitation relies on the hydrolysis of urea leading to ammonium and carbonate formation, the latter precipitating with available metals to form carbonates (Kumari et al. 2016). It has been shown that this process can be used to prepare pure specific metal carbonates and that some of the biominerals thus produced may exhibit nanoscale dimensions (Li et al. 2014, 2015). The aim of this research was to characterize nanoscale copper carbonate precipitated by ureolytic fungi and to investigate the influence of extracellular protein on the formation and morphology of such nanominerals.

## Materials and methods

### Organisms and media

The experimental fungi used were *Neurospora crassa* (WT FGSC no. 2489, Fungal Genetics Stock Centre (FGSC), Kansas, USA), *Pestalotiopsis* sp. and *Myrothecium gramineum* (isolated from calcareous soil; deposited in the Geomicrobiology Group Culture Collection) (Li et al. 2015). These were incubated on urea-modified AP1 agar plates at 25 °C in the dark for several days (3–4 days for *N. crassa* and *Pestalotiopsis* sp., 2 weeks for *M. gramineum*) prior to experimental subculture, and inoculation plugs were taken from the margins of actively growing colonies using a sterile (autoclaved at 121 °C, 15 min) cork borer (5 mm in diameter). All experiments were conducted at least in triplicate. Modified AP1 liquid medium was prepared according to Li et al. (2015).

### Identification of biominerals precipitated by fungal supernatants

Minerals precipitated by fungal growth supernatants were collected for further examination using an environmental scanning electron microscope (ESEM) (Jeol JSM7400F) and energy-dispersive X-ray analysis (EDXA). Experimental procedures were carried out as described by Li et al. (2015).

For mineral particles in the nanoscale, morphological observations were carried out using a Jeol-1200 EX transmission electron microscope (TEM) (Jeol, Welwyn Garden City, UK). About 10 mg of nanoscale particles was suspended in an Eppendorf tube containing 1 ml Milli-Q water, and 2 μl of the suspension was placed on “holey” carbon-coated nickel grids (Agar Scientific Ltd., Colchester, Essex, UK) and allowed to evaporate prior to TEM analysis.

FTIR was carried out for further identification of metal carbonates with sample preparation according to the procedure described in Li et al. (2015).

### Quantification of extracellular proteins produced by ureolytic fungi

Fungal growth supernatant was collected by centrifugation (4770 *g* × 20 min, 4 °C), and the concentration of extracellular protein was measured using the Coomassie (Bradford) Protein Assay Kit (Thermo Fisher Scientific Inc., Waltham, MA, USA). According to the protocol provided, 100 μl fungal growth supernatant was mixed well with 100 μl Coomassie reagent in a 96-well plate (Thermo Fisher Scientific Inc., USA) and left for 15 min. The absorbance was measured at 595 nm using a μQuant microplate spectrophotometer (BioTek Instruments, Inc., Winooski, VT, USA). Protein concentrations were estimated by reference to the absorbance obtained for a dilution series of bovine serum albumin (BSA) standards (Thermo Fisher Scientific Inc., USA).

### Geochemical modelling of copper carbonate precipitation using the Geochemist’s Workbench (GWB)

The Geochemist’s Workbench (GWB, 10.0.6) (Aqueous Solutions LLC, Urbana-Champaign, USA) is a set of software tools for manipulating chemical reactions, calculating stability diagrams and the equilibrium states of natural waters, tracing reaction processes, modelling reactive transport, plotting the results of these calculations and storing the related data. The GWB Essentials release contains six programs, GSS, Rxn, Act2, Tact, SpecE8 and Gtplot. For example, Act2 is a programme that calculates and plots activity-activity diagrams, which shows the stability of minerals and the predominant aqueous species in chemical systems. In this diagram, the axis variable can be species activity, gas fugacity, activity or fugacity ratio, pH, Eh or pe (electronic activity). SpectE8 models the equilibrium state of geochemical systems that contain an aqueous fluid, including the equilibrium distribution of aqueous species, the fluid’s saturation state with respect to minerals, the sorption of aqueous species onto various surfaces and the fugacity of gases dissolved in the fluid (https://www.gwb.com/Student/index.php).

## Results

### Geochemical modelling of copper carbonate precipitation using Geochemist’s workbench (GWB)

**Fig. 1:**
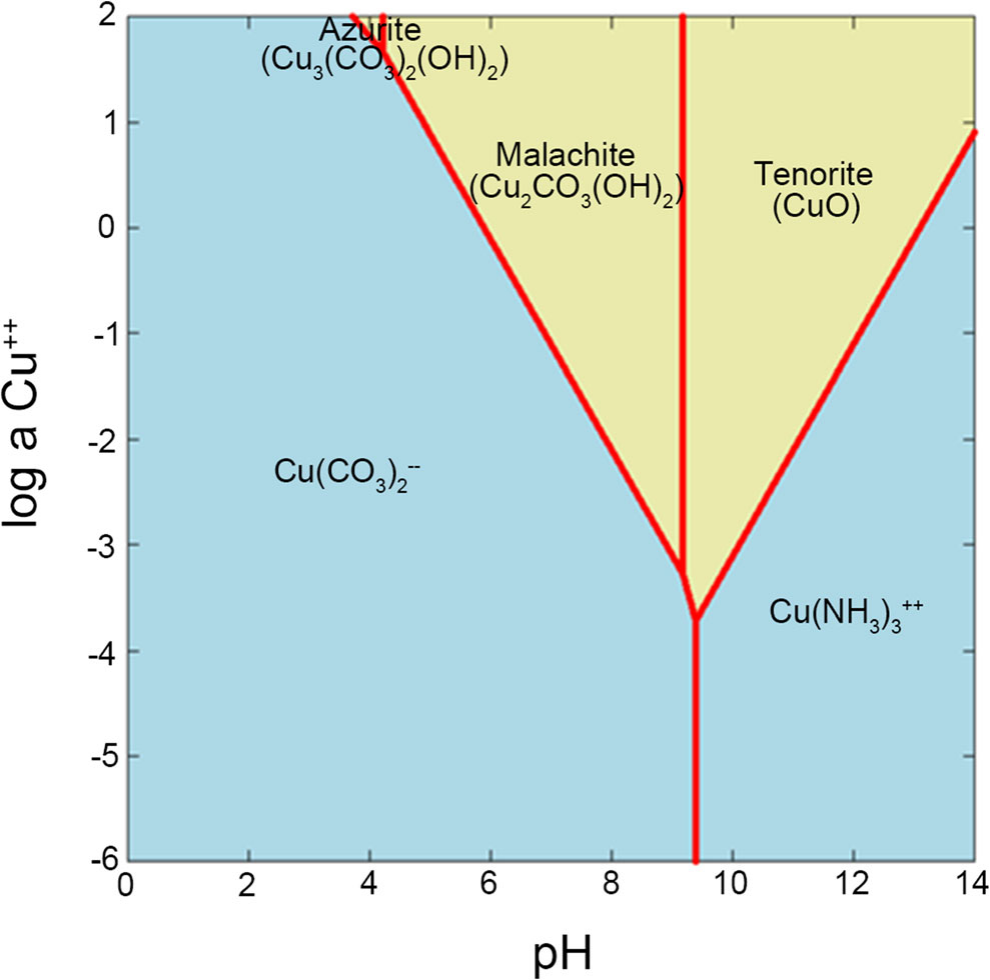
Solubility diagram of Cu^2+^ versus pH at 25 °C in a simulated fungal growth supernatant system. In this model, the solubility and stability of Cu^2+^ and relevant Cu-containing minerals and the predominance of aqueous Cu species were calculated individually in a simulated fungal growth supernatant system using GWB SpecE8 and Act2 programs. The simulated fungal supernatant system was set at 40 mM CO_3_^2−^, 80 mM NH_4_^+^, 4 mM KCl, 0.8 mM MgSO_4_·7H_2_O, 1.7 mM NaCl, 0.2 mM CaCl_2_·6H_2_O, 9 μM FeCl_3_·6H_2_O, 0.01 mM ZnSO_4_·7H_2_O and 0.02 mM MnSO_4_·4H_2_O at 25 °C

The Geochemist’s workbench (GWB) is a useful geochemical modelling software package for calculating activity-activity and temperature-activity diagrams, the equilibrium state of natural fluids and theoretical systems and reaction path modelling (Kaszuba and Runde 1999; Cleverley and Bastrakov 2005; Gallios and Vaclavikova 2008). Previous research has demonstrated that carbonate-laden fungal growth supernatants can provide a useful method for the precipitation of certain metal carbonates. The GWB software was applied to simulate the precipitation of copper carbonate in order to provide more theoretical understanding of the bioprecipitation of specific metal carbonates over a range of physico-chemical conditions. In these experiments, the solubility and stability of Cu^2+^ and relevant Cu-containing minerals and the predominance of aqueous Cu species were calculated individually in a simulated fungal growth supernatant system using the GWB SpecE8 and Act2 programs (Fig. 1). The simulated fungal supernatant system was set at 40 mM CO_3_^2−^, 80 mM NH_4_^+^, 4 mM KCl, 0.8 mM MgSO_4_·7H_2_O, 1.7 mM NaCl, 0.2 mM CaCl_2_·6H_2_O, 9 μM FeCl_3_·6H_2_O, 0.01 mM ZnSO_4_·7H_2_O and 0.02 mM MnSO_4_·4H_2_O at 25 °C. The results showed that the precipitation of different metal carbonates only occurred at certain metal and carbonate concentrations and pH range. At given physico-chemical conditions, several copper-containing species may occur in the aqueous system. The model diagram indicated that malachite (Cu_2_CO_3_(OH)_2_) might occur as the dominant mineral over the pH range of 4.2–9.2, at certain concentrations of Cu^2+^, while precipitation of tenorite (CuO) occurred at higher pH values (above pH 9). According to the solubility diagram, the precipitation of azurite (Cu_3_(CO_3_)_2_(OH)_2_) can only occur over a narrow pH range (pH ≈ 3.8–4.2) when the concentration of Cu^2+^ is unfeasibly high. In nature, malachite and azurite can be found on weathered brass, bronze copper (Schweitzer 2004). Azurite is unstable in the open air with respect to malachite, and often is pseudomorphically replaced by malachite:

2 Cu_3_(CO_3_)_2_(OH)_2_ + H_2_O → 3 Cu_2_(CO_3_)(OH)_2_ + CO_2_

### Characteristics of copper carbonate precipitated by fungal growth supernatants

**Fig. 2:**
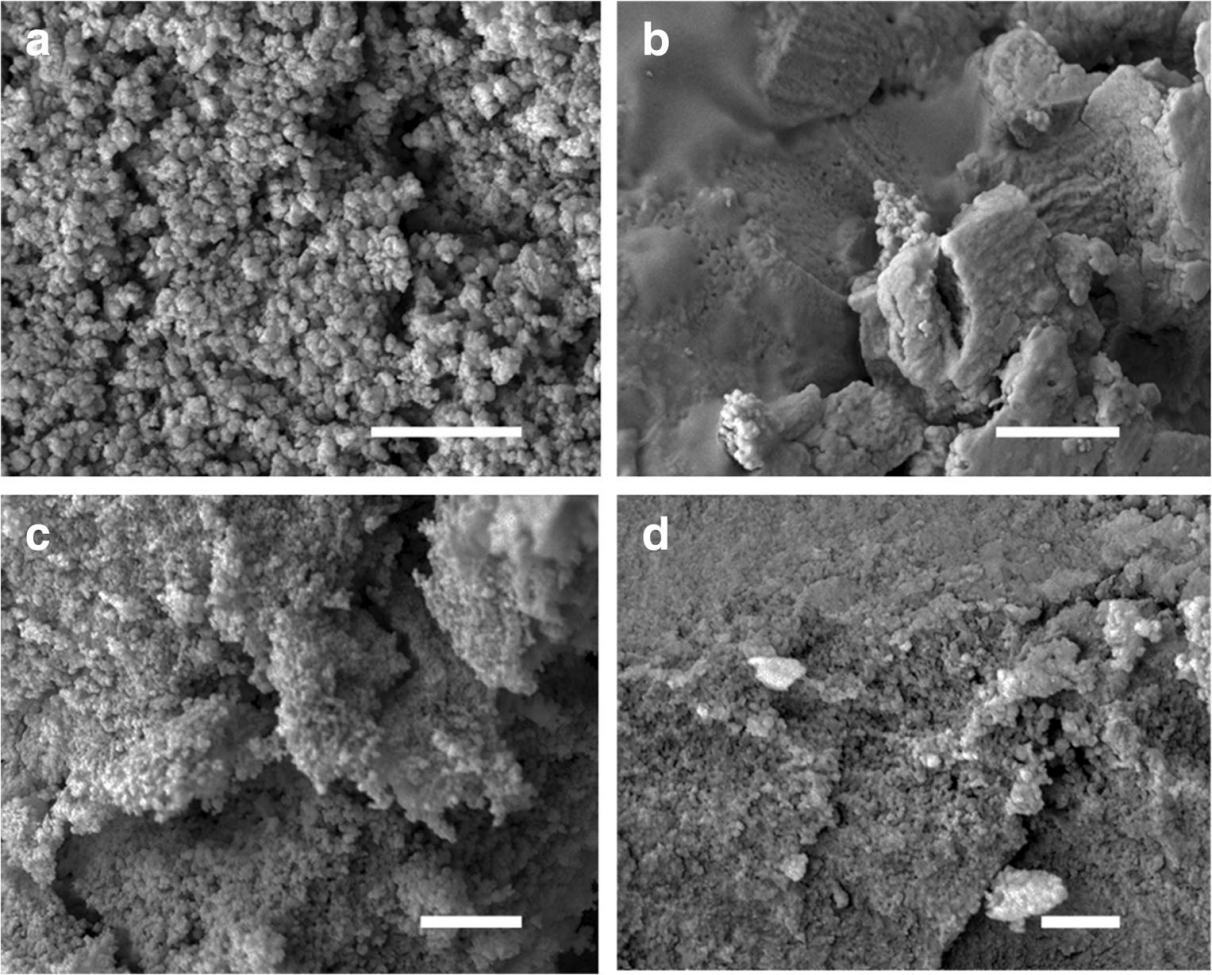
Scanning electron microscopy of minerals precipitated by a mixture of different fungal supernatants and CuCl_2_ solutions. Images show minerals precipitated by 50 mM CuCl_2_ when mixed with supernatants of **a** *N. crassa*, **b** *M. gramineum*, and **c**, **d** *Pestalotiopsis* sp. grown in modified AP1 medium. *Scale bars*: **a**–**c** = 1 μm, **d** = 500 nm. Typical images are shown from many similar examples

**Fig. 3:**
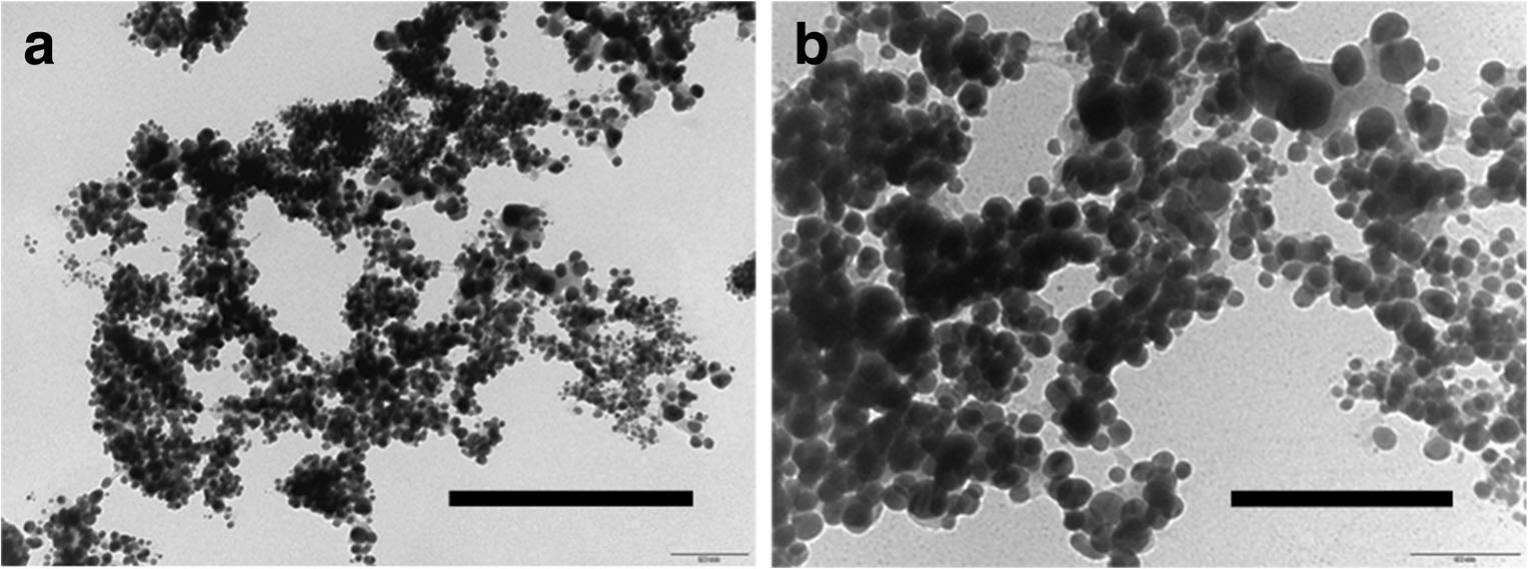
Transmission electron microscopy of minerals precipitated by mixture of CuCl_2_ solutions and the growth supernatant of *N. crassa*. *Scale bars*: **a** = 600 nm, **b** = 200 nm. Typical images are shown from many similar examples

**Fig. 4:**
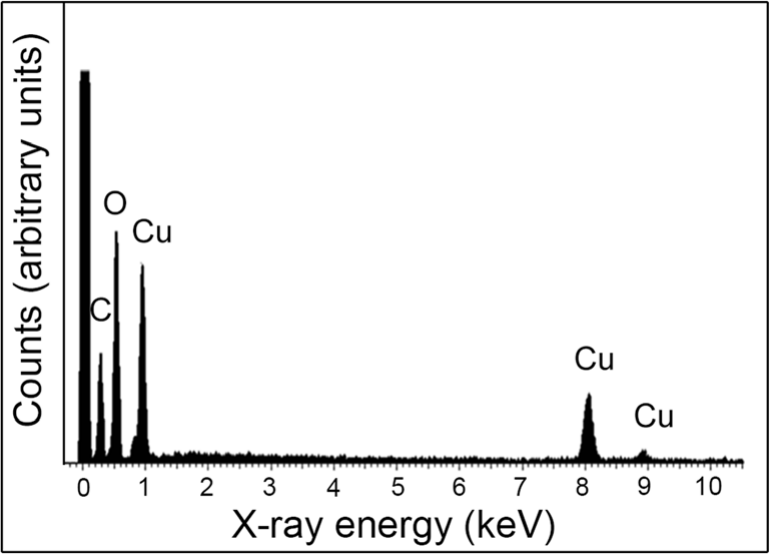
Energy-dispersive X-ray analysis of minerals precipitated by the supernatant of *N. crassa* when mixed with 50 mM CuCl_2_. Typical spectra are shown from one of several determinations

In these experiments, ureolytic fungi were grown in modified AP1 liquid media for several days at 25 °C (12 days for *N. crassa*, 14 days for *Pestalotiopsis* sp., 23 days for *M. gramineum*) and the fungal growth supernatant containing carbonate was collected by centrifugation (4770 *g* × 20 min, 4 °C). 50 mM CuCl_2_ was mixed with different fungal growth supernatants (*v*/*v* = 1:1) for the precipitation of metal carbonates. Minerals precipitated were collected by centrifugation (12,000 *g* × 5 min, 4 °C), washed twice by resuspending in Milli-Q and recentrifuging and then dried in a desiccator for a week at room temperature. The results showed that after mixture with the fungal growth supernatants, granular particles were produced and the dimensions of these were nanoscale (Fig. 2). TEM revealed that the minerals precipitated by *N. crassa* supernatant when mixed with CuCl_2_ were around 10–20 nm in diameter (Fig. 3). EDXA showed that the elemental composition of the minerals precipitated using the different fungal growth supernatants was the same in each case with the main elements being C, O and Cu (Fig. 4).

**Fig. 5:**
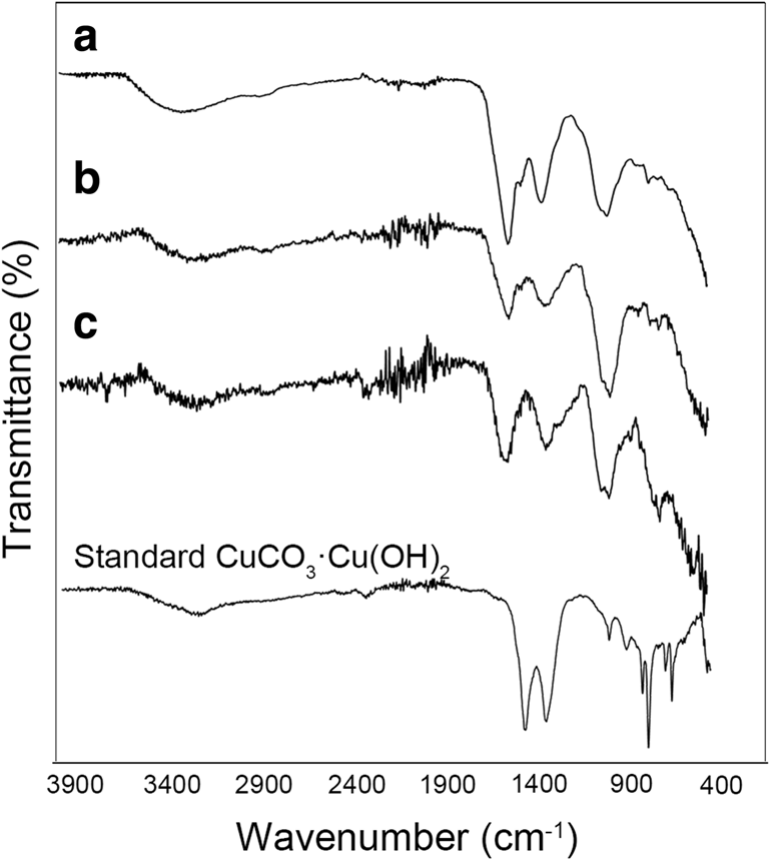
Fourier transform infrared spectroscopy of Cu-containing minerals precipitated by the fungal supernatants of **a** *N. crassa*, **b** *Pestalotiopsis* sp. and **c** *M. gramineum* when mixed with 50 mM CuCl_2_. Commercial basic copper carbonate (CuCO_3_·Cu(OH)_2_) was used as a standard. Typical spectra are shown from one of several determinations

FTIR was applied to assist the identification of Cu-containing minerals due to their amorphous pattern in X-ray diffraction (XRD) analysis. Commercial basic copper carbonate was used as a control for mineral identification (Fig. 5). Basic copper carbonate normally refers to the compound Cu_2_(OH)_2_CO_3_ (the mineral malachite). Sometimes, this name is also used for Cu_3_(OH)_2_(CO_3_)_2_ (the related mineral azurite). Table 1 shows the infrared spectra peaks and intensities of malachite and azurite (Huang and Kerr 1960) which provides information about the differences between these two minerals. According to Fig. 5, the main peaks of the control sample centred at ~ 3266, 1501, 1391, 953, 862, 834, 743 and 713 cm^−1^. Compared with the spectral data in Table 1, the commercial basic copper carbonate used should therefore be described as a mixture of malachite and azurite. Similarly, the Cu-containing minerals precipitated by the growth supernatants of *N. crassa* and *Pestalotiopsis* sp. were malachite with a trace amount of azurite while minerals precipitated by the supernatant of *M. gramineum* were solely malachite. Moreover, when the supernatant was mixed with CuCl_2_, the pH of the mixture decreased from pH 8–9 to pH 6 and, according to the solubility diagram, when the concentration of Cu^2+^ in the solution was ~ 50 mM, malachite (Cu_2_CO_3_(OH)_2_) was the dominant mineral over the pH range of 6 to 9.2 which agreed very well with the data from the FTIR results.

**Table 1:** Positions and intensities of absorption bands for copper carbonates using a PerkinElmer Model 21 double beam recording infrared spectrophotometer (adapted from Huang and Kerr 1960)

Minerals	Position of absorption bands (cm^−1^)									
Malachite (green) Cu_2_CO_3_(OH)_2_	3510 S		1510	1430 M	1099		873 M	822 M	750 W	712 W
				1395 S	1050 S			775 W		
Azurite (blue) Cu_3_(CO_3_)_2_(OH)_2_	3520 S	1852 W	1509	1422 S	1093 W	955 S	835 S	817 M	769 W	
			1475 W						743 W	

*W* weak, *S* strong, *M* medium

### Characteristics of commercial and chemically synthesized copper carbonates

**Fig. 6:**
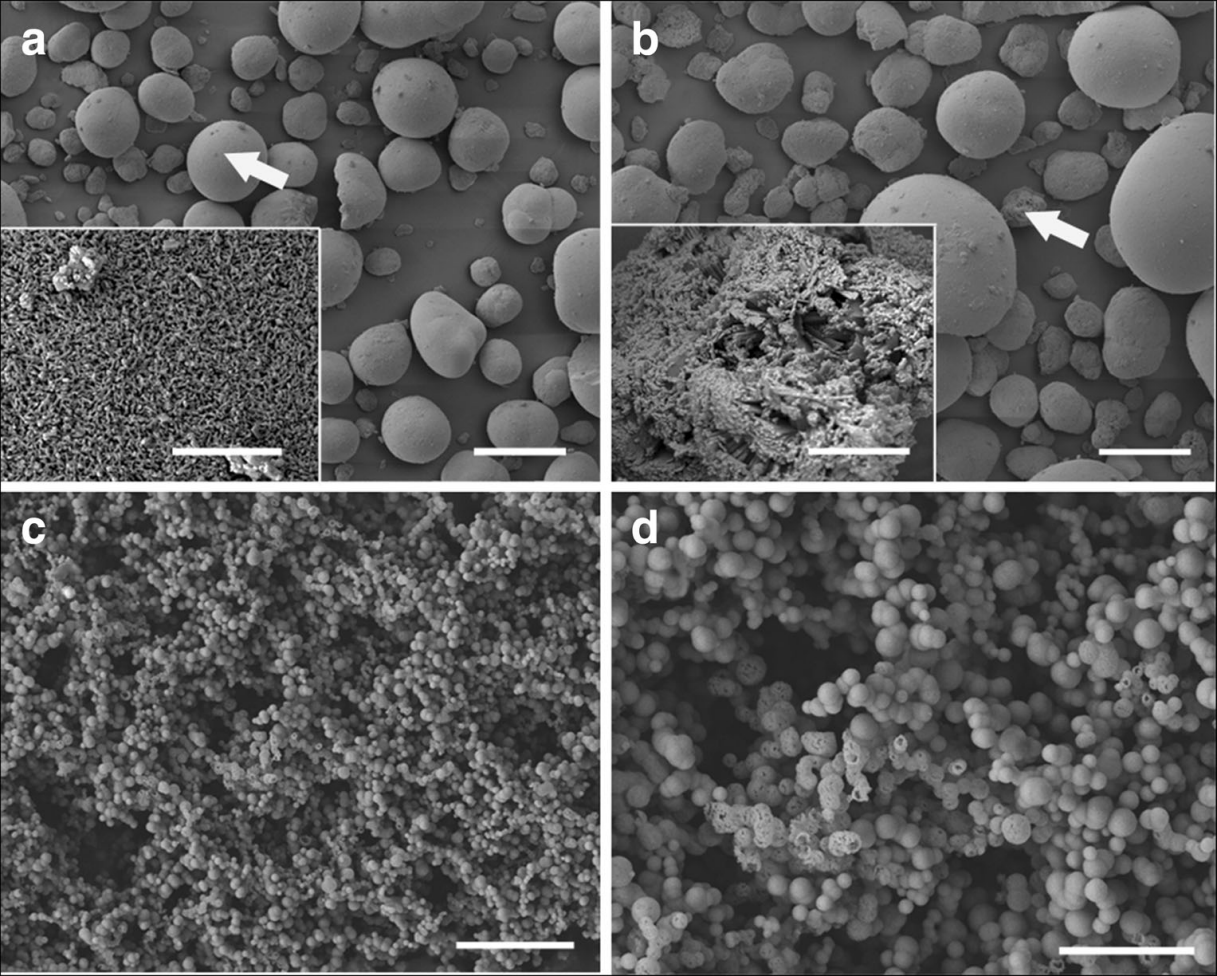
Scanning electron microscopy of **a**, **b** commercial basic copper carbonate and **c**, **d** copper carbonates precipitated by mixture of (NH_4_)_2_CO_3_ and CuCl_2_. *Scale bars*: **a** = 50 μm, *inset* = 2 μm; **b** = 30 μm, *inset* = 4 μm; **c** = 4 μm; **d** = 2 μm. Typical images are shown from many similar examples

Although sharing the same chemical formulae, minerals with different morphologies may show varying physical properties. To compare the differences between carbonate minerals synthesized using different methods, the morphology of commercial and chemically synthesized copper carbonate was investigated by SEM (Fig. 6). The morphology of commercial copper carbonate was found to be spherical with two different surfaces. One type of mineral contained compact short mineral columns which distributed evenly forming a smooth surface (Fig. 6a). The other type contained rod-shaped minerals which distributed loosely forming a rough surface (Fig. 6b). 50 mM CuCl_2_ was mixed with 50 mM (NH_4_)_2_CO_3_ (*v*/*v* = 1:1) for the precipitation of copper carbonate. The results showed that chemically synthesized copper carbonate was spherical with smooth or rough surfaces (~ 300 nm in diameter) (Fig. 6c, d).

### Extracellular proteins in fungal growth supernatants

Although the GWB software was applied as a useful method to predict and/or confirm the final products of the biomineralization process, little attention has been paid to the clear differences in morphology and size between biogenic minerals and commercial or chemically synthesized minerals. In these experiments, the possible role of extracellular protein was investigated in relation to the precipitation of nanoscale metal carbonates.

The Bradford method was used for the detection and quantification of fungal extracellular protein. The results showed that the highest concentration of extracellular protein produced by *M. gramineum* was about 24 μg ml^−1^ while proteins produced by *N. crassa* and *Pestalotiopsis* sp. were 14 and 9 μg ml^−1^, respectively (Table 2). After mixture with 50 mM CuCl_2_ and copper carbonate precipitation, the concentration of extracellular protein in the fungal supernatant decreased to different levels. Compared with the other two fungal species, more protein was removed (~ 80%) from the supernatant of *N. crassa* after mixing with CuCl_2_ solution, while for the supernatant of *M. gramineum*, only 23% of protein was removed (Table 2).

**Table 2:** Concentrations of extracellular protein produced by fungi and removal by copper carbonate precipitation

Fungal species	Total extracellular protein produced by fungi (μg ml^−1^)	Protein remaining in the supernatant (μg ml^−1^)	Proportion of protein removed by mineral precipitation (%)
*N. crassa*	14.25 ± 0.26	2.94 ± 0.09	80.2
*Pestalotiopsis* sp.	8.89 ± 0.12	2.07 ± 0.15	48.2
*M. gramineum*	24.11 ± 1.12	20.08 ± 0.25	23.9

All fungi were incubated in modified AP1 liquid medium at 25 °C in the dark for several days (12 days for *N. crassa*, 14 days for *Pestalotiopsis* sp. and 23 days for *M. gramineum*), and the supernatant was collected by centrifugation (4770 *g* × 20 min, 4 °C). 50 mM CuCl_2_ was mixed with the different fungal growth supernatants (*v*/*v* = 1:1) for the precipitation of copper carbonate. The quantification of extracellular protein was carried out before and after the mixture. Measurements were taken from at least three replicates, and the values indicate the standard error of the mean

### Mineral precipitation in the presence of fungal extracellular protein

**Fig. 7:**
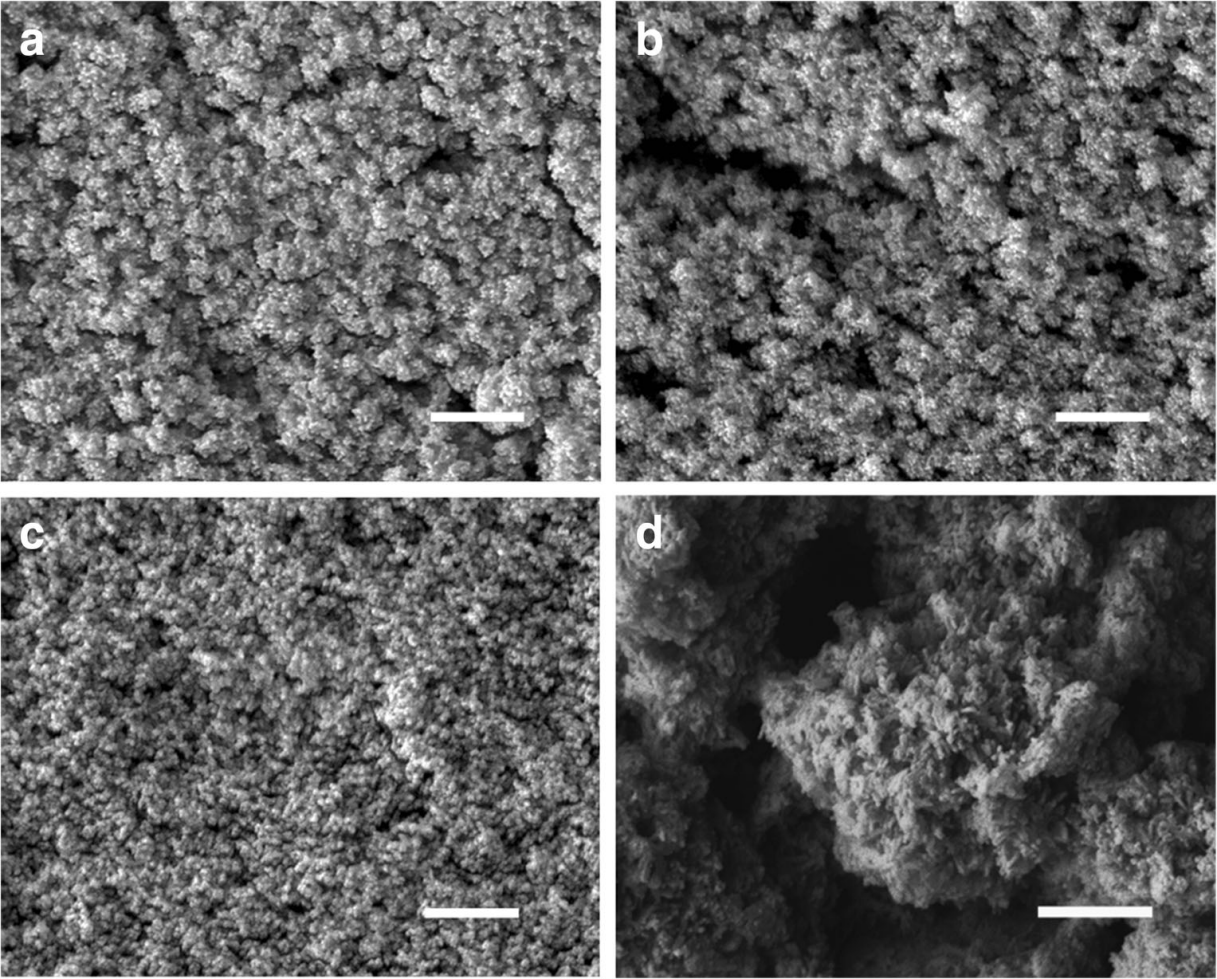
Scanning electron microscopy of Cu-containing minerals precipitated by mixture of (NH_4_)_2_CO_3_ and CuCl_2_ in the presence of proteins extracted from fungal growth supernatant of **a** *N. crassa*, **b** *Pestalotiopsis* sp., **c** *M. gramineum*, and **d** bovine serum albumin (BSA) standard solutions. *Scale bars* = 500 nm. Typical images are shown from many similar examples

To obtain pure and concentrated extracellular protein, fungal growth supernatants collected by centrifugation (4770 *g* × 30 min, 4 °C) were re-centrifuged (4770 *g* × 30 min, 4 °C) with a Vivaspin protein concentrator spin column (molecular weight cut-off 5000) (Sartorius Stedim Biotech, Göttingen, Germany) until the final volume of supernatant was around 2 ml. To investigate the role of extracellular protein on mineral morphology, standard bovine serum albumin (BSA) and concentrated extracellular protein (3.10 mg ml^−1^ for *N. crassa*, 1.22 mg ml^−1^ for *Pestalotiopsis* sp., 5.13 mg ml^−1^ for *M. gramineum*) diluted to an appropriate concentration with Milli-Q water were mixed with 50 mM CuCl_2_, and then added to 50 mM (NH_4_)_2_CO_3_ for the precipitation of metal carbonates. In the presence of fungal extracellular protein or BSA, all the minerals precipitated were in the nanoscale (Fig. 7). The minerals precipitated with addition of protein solution from *M. gramineum* were around 40 nm in diameter (Fig. 7c), almost three times smaller than the minerals precipitated with the addition of the other fungal proteins (Fig. 7a, b). Moreover, these minerals of smaller size formed spherical aggregates around 150 nm in diameter (Fig. 7a, b). In the presence of standard BSA, the minerals precipitated were around 35 nm in diameter which was similar to the minerals precipitated by proteins from *M. gramineum* (Fig. 7d).

After mixture, the concentration of protein in the solutions was measured and it was found that less protein was removed by the addition of (NH_4_)_2_CO_3_ to the protein solution (Table 3). More than 40% protein was removed from solution when added with CuCl_2_, except for the mixture containing BSA. When added with CuCl_2_ and (NH_4_)_2_CO_3_, little protein was detected in the mixture and more than 96% protein was removed (Table 3).

**Table 3:** Protein concentrations in different solutions and removal by the addition of CO_3_^2−^ and/or Cu^2+^

Protein source	Concentration of protein in solution (mg ml^−1^; proportion of protein removed from the solution, %)			
	Protein	Protein + CO_3_^2−^	Protein + Cu^2+^	Protein + Cu^2+^ + CO_3_^2−^
*N. crassa*	14.90 ± 0.33	14.38 ± 0.33 (3.5)	5.57 ± 0.17 (62.6)	0.46 ± 0.19 (96.9)
*Pestalotiopsis* sp.	8.39 ± 0.33	8.07 ± 0.12 (3.8)	5.03 ± 0.09 (40.0)	0.13 ± 0.05 (98.5)
*M. gramineum*	22.47 ± 1.71	22.02 ± 0.83 (2.0)	10.55 ± 0.78 (53.0)	0.58 ± 0.21 (97.4)
BSA	32.55 ± 0.67	30.99 ± 1.07 (4.8)	29.80 ± 0.61 (8.4)	0.36 ± 0.08 (98.9)

All fungi were incubated in modified AP1 medium at 25 °C in the dark for several days (12 days for *N. crassa*, 14 days for *Pestalotiopsis* sp. and 23 days for *M. gramineum*). To purify and obtain concentrated extracellular protein, fungal supernatant was centrifuged (4770 *g* × 30 min, 4 °C) with a Vivaspin protein concentrator spin column (molecular weight cut-off 5000). Concentrated extracellular proteins/standard BSA solutions were mixed with 50 mM (NH_4_)_2_CO_3_, and then added to 50 mM CuCl_2_ for precipitation of copper carbonates. Measurements were taken from at least three replicates, and the values indicate the standard error of the mean

## Discussion

Many techniques have been explored for the synthesis of nanomaterials, especially those using a biological template (Flynn et al. 2003; Royston et al. 2008; Shim et al. 2010; Sun et al. 2013). In comparison with materials produced by chemical and physical approaches, biominerals produced by biomineralization processes provide excellent physico-chemical characteristics as well as biological properties, which may, in part, be due to the various functional groups associated with organic substances (Ryu et al. 2010). In this work, we have investigated the production of nanoscale copper carbonate precipitated by fungal supernatants.

Ureolytic microorganisms have been shown to be promising candidates for the formation of metal carbonates (Ferris et al. 2004; Ghashghaei and Emtiazi 2013; Phillips et al. 2013; Li et al. 2014, 2015, 2016; Kumari et al. 2016). Previous research has demonstrated that *N. crassa* was particularly effective for precipitation of CaCO_3_ and over 90% of supplied calcium (50 mM) was precipitated as calcite (CaCO_3_) (Li et al. 2014). Such performance of urease-positive fungi incubated in urea-containing media provided an alternative technique for the synthesis of other metal carbonates. For example, ~ 50% of cadmium, supplied at a high concentration (0.5 M), was precipitated as pure otavite (CdCO_3_), with microscale or nanoscale structures, by culture supernatants obtained after growth of *N. crassa* in urea-supplemented medium (Li et al. 2014). Other urease-positive fungi (*Pestalotiopsis* sp. and *M. gramineum*), isolated from calcareous soil, were also applied for the precipitation of Ca and Sr carbonates, and the final minerals produced included CaCO_3_, SrCO_3_, Sr-containing vaterite ((Ca_x_Sr_1-x_)CO_3_) and the rare mineral olekminskite (Sr(Sr, Ca)(CO_3_)_2_) (Li et al. 2015). Moreover, fungal supernatants obtained after growth of ureolytic fungi in urea-modified media were also applied as an alternative method for the precipitation of other metal carbonates, with applications for the biorecovery of valuable metals, and preparation of electrochemically active metal oxides using the metal carbonate precursor (Li et al. 2014, 2015, 2016). In the experiments described here, different fungal growth supernatants were used for the precipitation of copper carbonates. The results showed that copper carbonates of various morphologies and sizes were precipitated after the mixture of different fungal growth supernatants and CuCl_2_ solutions. The precipitation process was simulated using GWB including parameters based on experimental data. The results showed that precipitation of copper carbonate strictly depended on the concentration of Cu^2+^ as well as the pH of the system. For example, at pH 8, copper carbonate precipitated only when the concentration of Cu^2+^ was above 3 mM. Thus, the solubility diagrams calculated according to the thermodynamic data within the GWB software can provide theoretical evidence for the precipitation of specific metal carbonates.

An organic matrix (protein and/or other biological macromolecules) can play an important role in certain biomineralization processes, regulating and controlling nucleation and growth of the inorganic structure (Aizenberg et al. 1996; Cha et al. 1999; Klaus et al. 1999; Kröger et al. 1999; Naik et al. 2002). Compared with commercial and chemically synthesized carbonates, a large proportion of the copper carbonates precipitated by the fungal growth supernatants showed a nanoscale character (the minimum size was around 10 nm), which may be related to the presence of extracellular protein in the growth supernatant. Several studies have demonstrated that proteins from living organisms can be employed as enzymes or templates for the synthesis of biominerals, even for minerals in the nanoscale (Douglas et al. 2002; Lee et al. 2002; Naik et al. 2002; Zhang 2003). Alexeev et al. (2003) showed that a ferric ion-binding protein from *Neisseria gonorrhoeae* readily bound clusters of Fe^3+^, Ti^4+^, Zr^4+^ or Hf^4+^ in solution which indicated a novel metal uptake mechanism and provided a model for protein-mediated biomineralization/dissimilation. Bovine serum albumin (BSA) was selected as a model protein for the synthesis of gold nanoclusters (NCs): prepared BSA-Au NCs were highly stable both in solution and in solid form (Xie et al. 2009). Naik et al. (2002) also described the in vitro biosynthesis of silver nanoparticles using silver-binding peptides identified from a combinatorial phage display peptide library. In our experiments, varying concentrations of extracellular protein were detected in the fungal growth supernatants depending on the organism and incubation time. When extracellular protein from fungal supernatants or standard BSA was mixed with CuCl_2_ and (NH_4_)_2_CO_3_, nanoscale Cu-containing minerals were produced and more than 96% of extracellular protein or BSA was removed from solution by the carbonate precipitation. In the presence of CuCl_2_, more than 40% of fungal extracellular protein was removed from the mixture. It is well known that different proteins or peptides show various affinities for different metal ions which is based on the interaction between an electron-donating group on a protein surface and the accessible coordination sites of the metal ion (Ueda et al. 2003). Amino acids such as histidine (Arnold 1991; Wong et al. 1991; Gutiérrez et al. 2007), tryptophan (Horrocks and Collier 1981) and cysteine (Hansen et al. 1996), are key contributors to protein-metal binding (Porath et al. 1975; Ueda et al. 2003). In our experiments, the metal ion (Cu^2+^)-extracellular protein complex might serve as a nucleation site, which then reacts with available CO_3_^2−^ in solution for the precipitation of metal carbonates. Such protein binding to the metal ions might limit the subsequent growth of minerals resulting in the formation of nanoscale minerals.

Compared with geological or abiotic counterparts, biogenic minerals often show different morphologies, crystal habits and material properties (Nudelman and Sommerdijk 2012). This is not only due to the ionic composition of the medium but also the specialized macromolecules such as polysaccharides and proteins which can act as promoters or inhibitors of crystal nucleation, growth and phase transformations (Addadi and Weiner 1985; Addadi et al. 1987; Falini et al. 1996; Weiner and Addadi 1997; Nudelman and Sommerdijk 2012). This research has demonstrated that fungal metabolites, especially extracellular protein, can play an important role in the precipitation and morphology of metal carbonates, providing a potentially useful approach for the synthesis of specific metal carbonate nanoparticles. It is also concluded that geochemical modelling of metal speciation and mineral precipitation can provide useful theoretical support to identify physico-chemical conditions for optimal synthesis of desired nanoscale metal carbonates.
